# Phenotypical characterization of the peripheral blood T cells in patients with celiac disease: does it differentiate suspicious celiac disease cases?

**Published:** 2015

**Authors:** Hadi Hossein Nataj Arab, Mohsen Masjedi, Fereshteh Alsahebfosoul, Mojgan Mokhtari, Nahid Jamali, Mohammad Hassan Emami, Ali Saffaei

**Affiliations:** 1 *Mazandaran University of Medical Sciences, Sari, Iran*; 2 *Isfahan University of Medical Sciences, Isfahan, Iran*; 3 *Poursina Hakim Research Center, Isfahan, Iran*; 4 *Iranian Celiac Association, Isfahan, Iran*; 5 *Pharmacy Students’ Research Committee, School of Pharmacy, Isfahan University of Medical sciences, Isfahan, Iran *

**Keywords:** Celiac disease, T-Lymphocytes, Blood, Flow cytometry, Iran

## Abstract

**Aim:**

The present study aimed to study the immunological changes seen in the intestinal epithelium of the celiac patients could also be detected in the peripheral blood lymphocyte populations.

**Background:**

Celiac disease (CD) is a small bowel enteropathy caused by permanent wheat gluten intolerance. One of the earliest signs of CD is an increase in the numbers of the intestinal intraepithelial lymphocytes (iIEL).

**Patients and methods:**

In this case-control study, totally 13 untreated subjects with acceptable criteria for CD without any complication and 16 healthy subjects without any positive criteria for CD were selected. Peripheral blood T cells were analyzed by two-color flow cytometry in both groups.

**Results:**

The mean age of patients was 33.6 ± 3.4 years and two patients had Marsh IIIB, five patients had Marsh IIIA and six patients had Marsh II histology class. The mean percentages of the TCR^+^ T cells in the patients were significantly higher than the controls (*p*=0.015). However, the mean percentages of the αβTCR^+ ^T cells were significantly lower in the untreated patients than the controls (*p*=0.025). There were no significant difference between the mean percentages of lymphocytes expressing the CD3, CD4 and CD8 molecules in the patients and the controls.

**Conclusion:**

The change in the percentages of the peripheral blood T cells expressing the γδTCR and αβTCR in the celiac patients could be used in conjunction with the other serological markers to identify new CD cases.

## Introduction

 Celiac disease (CD) is defined as an intolerance of the small bowel to gluten (1), whereas clinical and histological improvement is seen after withdrawal of gluten from the diet (2, 3). The pathogenesis of CD is thought to involve a cell-mediated mucosal immune response in genetically susceptible subjects (4, 5). The HLA DQ2 heterodimer confirms genetic susceptibility to CD; the DQ2 allele is carried by nearly 90% of the patients. The remainder carries the HLA DQ8 allele (-).

Celiac disease is heterogeneous in its clinical presentation (10, 11). There is also a latent form of the disease (12, 13), that describes those subjects suspected of having the disease with a positive antibody blood test, but normal duodenal histology while eating a normal gluten-containing diet (14, 15). Increased densities of total intestinal intraepithelial lymphocytes (iIEL) (-) and particularly iIEL expressing the γδTCR have been reported in both untreated and treated CD. Interestingly, increased iIEL counts have also been observed in the duodenal mucosa from genetically susceptible healthy subjects (latent CD) (-). The mechanism action of the γδTCR^+ ^iIEL in CD is not fully understood, but this subset of iIEL with the cytotoxic action could play an essential role in the intestinal epithelium destructive procedure. Nevertheless, the increased numbers of the γδTCR^+^ iIEL in CD appear to be dependent on the dietary treatment. In addition, it has been proposed that the αβTCR^+ ^iIEL are sensitized to the gliadin and play an essential role in the pathogenesis of the villous atrophy (24, 25). 

In addition, lamina propria T cells in celiac lesions show signs of activation, as they express the CD25 (IL-2R^+^) activation marker. This is also the case in the duodenal mucosa obtained from genetically susceptible healthy subjects (26). These observations suggest that subtle immunological changes have already occurred in the duodenal mucosa of subjects with latent CD prior to the onset of clinical disease. In view of the findings reported in the mucosal biopsies from CD patients, this study aimed to measure the percentages of the peripheral blood γδTCR^+^ T cells in the CD patients. Since increased densities of the γδ TCR^+^ iIELs are found in the early stages of celiac entropathy when the mucosal morphology is still normal, we hypothesized that the elevation of circulating γδTCR^+^ T cells could be an immunological hallmark that could be used in the diagnosis of CD. 

## Patients and Methods

***Patients***

In this case-control study, thirteen untreated patients (8 females, 5 males, mean age 33.6 years, SD 3.4 years) with untreated CD who referred to the Al-Zahra hospital or Poursina Hakim Research Center, Isfahan, Iran, were studied. Patients who had other complications, previous gastrointestinal surgery, unknown disease of the gastrointestinal region, well-known gastrointestinal malignancy and chronic diseases were excluded. The sample size was determined based on the previous studies in Isfahan, Iran by Emami *et al.* (27). Diagnosis was made based on the presence of clinical presentations, CD-related antibodies, and conventional Marsh histopathological classification. All patients underwent upper gastrointestinal endoscopy. A “Pentax EPM-3300” EG2940 scope was used for the endoscopy. The second part of duodenum was observed and four spike biopsies were reserved from the second part of duodenum. Biopsy samples were fixed in 10% fresh buffered formalin and embedded in paraffin and finally cut in 5 μm pieces and stained with hematoxylin-eosin. The Ethical Committee of the Isfahan University of Medical Sciences, and the Iranian Celiac Association approved the study protocol, and the participants gave written informed consent after explaining the purposes and protocol of the study.

***Controls***

Controls were 16 gender-matched healthy subjects (10 female, 6 male, and aged range 15 to 79 years) who were negative for CD-specific serum IgA anti-endomysium and anti-tTG antibodies. All healthy controls underwent an upper GI endoscopy, with biopsy of the duodenum (D2). All control subjects had normal endoscopic appearance at D1 and D2, and normal histology of the D2 biopsy. 

***Estimation of antibodies and lymphocytes***

Peripheral blood was collected from the CD patients at the time of diagnosis before the commencement of a GFD, and also from the controls. Serum IgA anti-endomysium and anti-tissue transglutaminase (anti-tTG) antibodies (IgA and IgG) were measured using the ELISA assay. Lymphocyte subsets in anti-coagulated (EDTA) peripheral blood of patients and controls were analyzed by two color direct immunofluorescence, using a lysed whole-blood technique. Lymphocyte subsets were examined within 4 hr of blood collection.

***Monoclonal antibodies***

The following commercial monoclonal antibodies were used: anti-CD3 conjugated to R. Phycoerythrin (RPE) (clone: UCHT1, purchased from the Serotec Company), anti-CD4 (RPE) (clone: RPA-T4, purchased from the Serotec Company), anti-CD8 conjugated to Fluorescein Isothiocyanate (FITC) (clone: Bu88, purchased from the Serotec Company), anti-αβTCR (FITC) (clone: IP26, purchased from the Serotec Company), anti- TCR (FITC) (Clone: 11F2, purchased from the Becton-Dickinson Company). 

***Labeling blood lymphocytes***

100 µl of anti-coagulated blood was incubated with a mixture of direct labeled monoclonal antibodies. 20 µl of the monoclonal antibody from the Becton Dickinson Company or 10 µl of the monoclonal antibody from the Serotec Company were used. After 15 minutes of incubation at room temperature in the dark, erythrocytes were lysed by addition 100 µl of lysis solution (IQ Lyse, IQ Product, and Netherlands) and incubated for 10 minutes in the dark. 2 ml of distilled water was then added and incubation continued for a further 10 minutes. Following centrifugation and washing in PBS, the cells were resuspended in PBS containing 1% paraformaldehyde and kept at 4° C until analysis.

***Phenotypic analysis of lymphocytes by the flow cytometry***

Two-color flow ctometric analysis was carried out using a FACScan (Becton Dickenson, USA), with Cell Quest and Win MDI 2.9 software. The lymphocyte population was identified by a combination of forward, side scatter and immunofluorescence. Calibrate beads (Becton Dickinson, USA) were used for the instrument set-up and the fluorescent compensations were adjusted using a mixture of PE-conjugated anti-CD4 and, FITC-conjugated anti-CD8. FITC and PE conjugated monoclonal antibodies were used for control staining and gates were set to exclude with fluorescence intensity less than 99.9% of the appropriate controls. Background fluorescence was determined with the FITC-conjugated IgGl, PE-conjugated IgG1, and subtracted from the results. Fluorescence from FL1 (FITC) and FL2 (PE) channels were utilized to measure the cell surface antigens. Percentages of cells showing positive fluorescence for a particular marker were calculated from dual parameter displays of fluorescence from channels FL1 (FITC) and FL2 (PE). The results were expressed as the mean percentages of the CD3^+^ cells, CD4^+^ cells, CD8^+^ cells and T cells that express the αβTCR and γδTCR markers.

***Statistical analysis***

Analysis of data was performed using the FACSCalibur flow cytometer and Cell Quest software. A paired-samples *t*-test was used to analyze the significance of the difference in the mean percentages of CD3^+^ cells, CD4^+^, CD8^+^, αβTCR^+^ and γδTCR^+^ T cells in the celiac patients and controls, using an independent-sample *t*-test to compare the percentages of the above cells in the celiac patients and controls. The analysis was performed using the SPSS software version 16.0 for windows. P-values 0.05 were considered significant. 

## Results

Duodenal biopsies revealed that two patients had Marsh IIIB, five patients had Marsh IIIA and six patients had Marsh II histology class. 

**Table 1:** The percentages of the different T cell subsets in the peripheral blood of untreated celiac patients and healthy controls. Values are expressed as mean ± SE

		**Cases**		**Controls**	***p*** **-value** ^1^	**Significance status**
% / T cells	CD3^+^	72.81 ± 1.94	7.64 ± 2.84		0.557	NS^2^
	CD4^+^	39.91 ± 1.87	44.16 ± 1.25		0.064	NS
	CD8^+^	25.37 ± 0.92	25.18 ± 1.37		0.918	NS
	CD4/CD8 ratio	1.60 ± 0.12	1.83 ± 0.42		0.198	NS
	αβTCR^+^	89.74 ± 1.05	93.64 ± 0.62		0.003	S^3^
	γδTCR^+^	7.58 ± 1.15	4.44 ± 0.52		0.014	S

1 *P*0.05 considered significant;

2 NS: Not significant;

3 S: Significant

**Figure 1:**
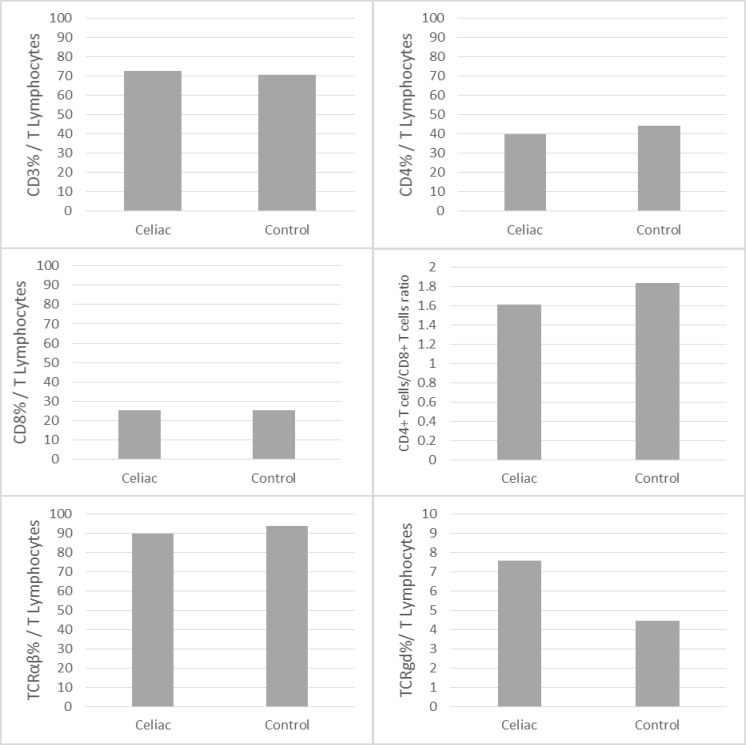
Frequency of the αβTCR and γδTCR expression on the T cells, CD3^+^, CD4^+^, CD8^+^, CD4^+^/CD8^+^ ratio T lymphocyte subsets in the peripheral blood of the celiac patients and controls

**Figure 2:**
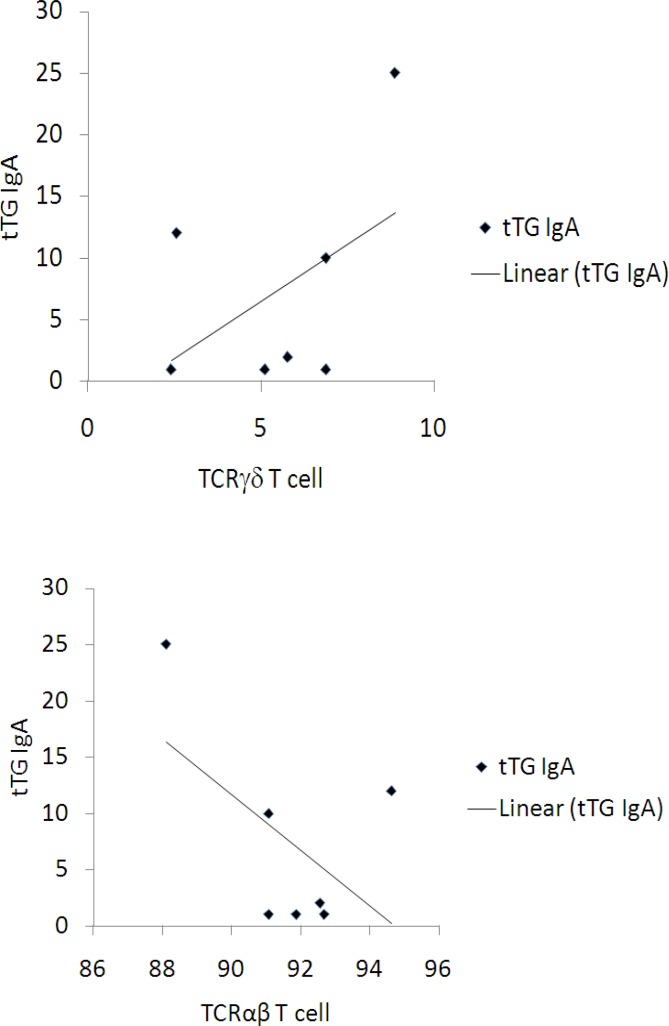
Correlation between the percentages of the peripheral blood   TCR^+^ T cell and concentration of tTG IgA and discrepancy between the percentages of the peripheral blood αβTCR^+^ T cells and concentration of tTG IgA.

On the other hand, two patients had flat small bowel mucosa and five had mild villous atrophy with the crypt hyperplasia consistent with CD. Subsequently, they all showed normalization of the duodenal mucosal morphology on the gluten free diet (GFD). 

Furthermore, the proportions of blood lymphocytes expressing T cell markers and ratios of CD4/CD8 T cells are summarized in table 1 and figure 1. The Kolmogorov–Smirnov test shows the distributions of data are normal (*p*>0.05). The levels of CD3^+^, CD4^+^ and CD8^+^ T cells weren’t equal, but there were no significant differences in the percentages of these cells in the CD patients and healthy controls (Table 1). The ratio of CD4^+^/CD8^+^ T cells (helper/suppressor ratio) was also calculated, and we found no significant difference between untreated CD patients and healthy controls (1.60 ± 0.12 *vs.* 1.83 ± 0.42; P=0.198). However, the mean percentages of the circulating TCR^+^ T cells in the patients were significantly higher than the controls (7.58 ± 1.15% *vs. *4.44 ± .052%; *p* = 0.014). Conversely, the mean percentages of the circulating αβTCR^+ ^T cells were significantly lower in the untreated patients than the controls (89.74 ± 1.05% *vs.* 93.64 ± 0.62%, P = 0.003). 

Furthermore, all 13 patients were positive for both the CD-specific serum IgA class EMA and tTG antibodies. The results also revealed slight correlation between the tTG IgA levels and the immune phenotype including the prevalence of the circulating TCR^+ ^T cells in the celiac patients (r=0.47) (figure 2). We also found a slight correlation between the diminution prevalence of the circulating αβTCR^+ ^T cells and the tTG IgA levels in the celiac patients (r=0.54). 

## Discussion

The current study aimed to determine the proportions of T cell subsets in the peripheral blood from the celiac patients and healthy controls in the Isfahan, Iran. There is little information available world-wide on the peripheral blood T cell subsets in the celiac patients. Some studies have shown that there are no differences in the subsets of the circulating CD3^+^ αβTCR^+^ T cells compared with healthy control subjects (25, 28).

We also found no significant differences in the mean percentages of the peripheral blood CD3^+^ T cells expressing the CD4 and CD8 markers between CD patients and healthy controls in Isfahan. Our findings are consistent with the findings of Lähteenoja *et al. *(29), and Kemppainen *et al.* (30) who reported the equal levels of the circulating CD3^+^, CD4^+^ and CD8^+^ T lymphocytes in the untreated CD patients and controls.

We found that patients from Isfahan with untreated CD had lower percentages of the peripheral blood T cells that expressed the αβTCR, compared to healthy controls. A possible reason for this difference is the greater numbers of T cells which express the αβTCR that are found in the intestinal epithelium of untreated celiac patients. These αβTCR^+^ iIEL may represent cells that have been 'sequestered' in the epithelium, leading to a decrease in the percentage of the circulating αβTCR^+^ T cells. Our findings differ from those of Kertulla *et al*., (24), who observed the equal proportions of the circulating αβTCR^+^ T cells in the untreated CD patients and controls. The difference between the studies may reflect the geographical region and the genetic background of the subjects, as these factors are known to affect the frequency of the circulating T cells expressing the αβTCR.

This study also revealed increased percentages of the circulating T cells expressing the TCR in the celiac patients compared with the healthy controls. The discrepancy between the mean percentages of the circulating T cells expressing the γδTCR in the untreated celiac patients and controls is probably due to a decrease in the percentages of circulating T cells expressing the αβTCR and migration of the intestinal TCR^+^ iIEL into the circulation.

The second phase of this study was to evaluate the correlation of the prevalence of peripheral blood T cells expressing the TCR and αβTCR with the serology test on the complete resolution of clinical signs and symptoms. Interestingly, we observed a correlation between the positive serum levels of tTG IgA and the prevalence of T cells expressing TCR and αβTCR, indicating a relation between these two phenomenons. This correlation has not been demonstrated in any previous studies (31). One study found a correlation between the serum levels of tTGgA and the prevalence of CD4^+^ and CD8^+^ T lymphocytes in GFD (32), but this study did not observe this correlation owing to the low sample size. The limitations of this study were the low sample size as well as focusing study to Isfahan city population in Iran. Nevertheless, the advantages of this study were the determination of the proportions of the circulating T cells expressing the γδTCR and αβTCR, and these findings may detect latent as well as histologically confirmed cases of CD. 

In conclusion, different studies show different percentages of the peripheral blood T cells in the CD patients and controls. This finding could be due to the environmental factors, sample sizes, genetic background and stage of disease. Further studies are required to look at these factors. More importantly, the proportions of the peripheral blood T cells expressing the γδTCR and αβTCR marker may detect latent as well as histologically confirmed cases of CD.
